# Quinoxalin-2-yl *p*-tolyl ether

**DOI:** 10.1107/S1600536808026834

**Published:** 2008-08-23

**Authors:** Nor Duha Hassan, Hairul Anuar Tajuddin, Zanariah Abdullah, Seik Weng Ng

**Affiliations:** a Department of Chemistry, University of Malaya, 50603 Kuala Lumpur, Malaysia

## Abstract

The dihedral angle between the two aromatic ring systems in the title compound, C_15_H_12_N_2_O, is 42.6 (1)°. The angle at the O atom is widened to 117.7 (1)°.

## Related literature

The title compound exhibits fluorescence; see: Abdullah (2005 ▶); Kawai *et al.* (2001 ▶); Mohd Salleh *et al.* (2007 ▶).
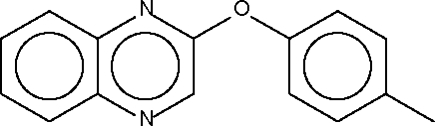


## Experimental

### 

#### Crystal data

C_15_H_12_N_2_O
*M* *_r_* = 236.27
Triclinic, 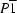

*a* = 5.2655 (1) Å
*b* = 9.1713 (2) Å
*c* = 12.8112 (2) Å
α = 74.660 (1)°
β = 81.163 (1)°
γ = 89.095 (1)°
*V* = 589.35 (2) Å^3^
*Z* = 2
Mo *K*α radiation
μ = 0.09 mm^−1^
*T* = 100 (2) K
0.25 × 0.25 × 0.25 mm

#### Data collection

Bruker SMART APEX diffractometer
Absorption correction: none
2828 measured reflections
2030 independent reflections
1764 reflections with *I* > 2σ(*I*)
*R*_int_ = 0.012

#### Refinement

*R*[*F* ^2^ > 2σ(*F* ^2^)] = 0.035
*wR*(*F* ^2^) = 0.099
*S* = 0.99
2030 reflections
164 parameters
H-atom parameters constrained
Δρ_max_ = 0.15 e Å^−3^
Δρ_min_ = −0.23 e Å^−3^

### 

Data collection: *APEX2* (Bruker, 2007 ▶); cell refinement: *SAINT* (Bruker, 2007 ▶); data reduction: *SAINT*; program(s) used to solve structure: *SHELXS97* (Sheldrick, 2008 ▶); program(s) used to refine structure: *SHELXL97* (Sheldrick, 2008 ▶); molecular graphics: *X-SEED* (Barbour, 2001 ▶); software used to prepare material for publication: *publCIF* (Westrip, 2008 ▶).

## Supplementary Material

Crystal structure: contains datablocks global, I. DOI: 10.1107/S1600536808026834/bt2771sup1.cif

Structure factors: contains datablocks I. DOI: 10.1107/S1600536808026834/bt2771Isup2.hkl

Additional supplementary materials:  crystallographic information; 3D view; checkCIF report

## Figures and Tables

**Fig. 1.**
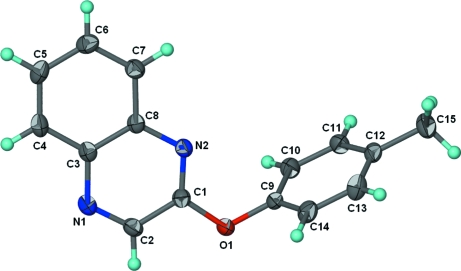
Displacement ellipsoid plot (Barbour, 2001) plot of C15H12N2O at the 70% probability level. Hydrogen atoms are drawn as spheres of arbitrary radius.

## supplementary crystallographic information

### Comment

(type here to add)

### Experimental

*p*-Cresol (0.54 g, 5 mmol) was dissolved in a small volume of water containing potassium hydroxide (0.20 g, 5 mmol). The mixture was heated to remove the water to give a brown compound. The compound and 2-chloroquinoxaline (0.82, g, 5 mmol) were heated in THF (15 ml) for 8 h. The mixture was in 1 N sodium hydroxide; the aqueous solution was extracted with dichloromethane. The organic phase was dried over sodium sulfate. Evaporation of the solvent gave a yellow product, which was was washed with chloroform to remove impurities. Crystals were obtained upon recrystallization from an ethyl acetate/hexane mixture.

### Refinement

Carbon-bound H-atoms were placed in calculated positions (C—H 0.95–0.98 Å) and were included in the refinement in the riding model approximation, with *U*(H) fixed at 1.2–1.5*U*(C).

### Crystal data

**Table:**

C_15_H_12_N_2_O	*Z* = 2
*M**_r_* = 236.27	*F*_000_ = 248
Triclinic, *P*1	*D*_x_ = 1.331 Mg m^−^^3^
Hall symbol: -P 1	Mo *K*α radiation λ = 0.71073 Å
*a* = 5.2655 (1) Å	Cell parameters from 2687 reflections
*b* = 9.1713 (2) Å	θ = 2.3–28.3º
*c* = 12.8112 (2) Å	µ = 0.09 mm^−^^1^
α = 74.660 (1)º	*T* = 100 (2) K
β = 81.163 (1)º	Irregular block, colorless
γ = 89.095 (1)º	0.25 × 0.25 × 0.25 mm
*V* = 589.35 (2) Å^3^	

### Data collection

**Table:**

Bruker SMART APEX diffractometer	1764 reflections with *I* > 2σ(*I*)
Radiation source: fine-focus sealed tube	*R*_int_ = 0.012
Monochromator: graphite	θ_max_ = 25.0º
ω scans	θ_min_ = 2.3º
Absorption correction: None	*h* = −6→6
2828 measured reflections	*k* = −7→10
2030 independent reflections	*l* = −15→15

### Refinement

**Table:**

Refinement on *F*^2^	Secondary atom site location: difference Fourier map
Least-squares matrix: full	Hydrogen site location: inferred from neighbouring sites
*R*[*F*^2^ > 2σ(*F*^2^)] = 0.036	H-atom parameters constrained
*wR*(*F*^2^) = 0.099	*w* = 1/[σ^2^(*F*_o_^2^) + (0.0589*P*)^2^ + 0.1439*P*] where *P* = (*F*_o_^2^ + 2*F*_c_^2^)/3
*S* = 0.99	(Δ/σ)_max_ = 0.001
2030 reflections	Δρ_max_ = 0.15 e Å^−^^3^
164 parameters	Δρ_min_ = −0.23 e Å^−^^3^
Primary atom site location: structure-invariant direct methods	Extinction correction: none

### Fractional atomic coordinates and isotropic or equivalent isotropic displacement parameters (Å^2^)

**Table:**

	*x*	*y*	*z*	*U*_iso_*/*U*_eq_	
O1	1.26514 (16)	1.02668 (10)	0.14690 (7)	0.0230 (2)	
N1	1.07057 (19)	0.73809 (12)	0.03457 (8)	0.0218 (3)	
N2	0.91228 (18)	0.86256 (11)	0.21386 (8)	0.0189 (2)	
C1	1.1133 (2)	0.91123 (13)	0.14121 (10)	0.0188 (3)	
C2	1.1953 (2)	0.84960 (14)	0.05056 (10)	0.0216 (3)	
H2	1.3450	0.8912	0.0006	0.026*	
C3	0.8547 (2)	0.68201 (13)	0.10938 (10)	0.0192 (3)	
C4	0.7105 (2)	0.56145 (14)	0.09688 (10)	0.0232 (3)	
H4	0.7628	0.5188	0.0373	0.028*	
C5	0.4948 (2)	0.50557 (14)	0.17061 (11)	0.0245 (3)	
H5	0.3980	0.4242	0.1619	0.029*	
C6	0.4161 (2)	0.56796 (14)	0.25901 (10)	0.0237 (3)	
H6	0.2654	0.5290	0.3093	0.028*	
C7	0.5546 (2)	0.68464 (14)	0.27340 (10)	0.0217 (3)	
H7	0.5005	0.7252	0.3339	0.026*	
C8	0.7766 (2)	0.74447 (13)	0.19880 (10)	0.0181 (3)	
C9	1.1976 (2)	1.09150 (13)	0.23540 (10)	0.0199 (3)	
C10	0.9936 (2)	1.18709 (14)	0.23647 (10)	0.0232 (3)	
H10	0.8934	1.2083	0.1788	0.028*	
C11	0.9373 (2)	1.25183 (14)	0.32362 (10)	0.0229 (3)	
H11	0.7965	1.3177	0.3252	0.028*	
C12	1.0819 (2)	1.22244 (13)	0.40840 (10)	0.0212 (3)	
C13	1.2913 (2)	1.12925 (14)	0.40226 (11)	0.0259 (3)	
H13	1.3958	1.1102	0.4584	0.031*	
C14	1.3511 (2)	1.06353 (14)	0.31607 (11)	0.0241 (3)	
H14	1.4953	1.0004	0.3127	0.029*	
C15	1.0175 (3)	1.29050 (15)	0.50378 (10)	0.0292 (3)	
H15A	0.8638	1.3514	0.4951	0.044*	
H15B	1.1620	1.3549	0.5063	0.044*	
H15C	0.9849	1.2095	0.5720	0.044*	

### Atomic displacement parameters (Å^2^)

**Table:**

	*U*^11^	*U*^22^	*U*^33^	*U*^12^	*U*^13^	*U*^23^
O1	0.0225 (4)	0.0229 (5)	0.0243 (5)	−0.0055 (4)	0.0043 (4)	−0.0114 (4)
N1	0.0235 (5)	0.0231 (6)	0.0198 (5)	0.0028 (4)	−0.0031 (4)	−0.0081 (4)
N2	0.0203 (5)	0.0176 (5)	0.0190 (5)	0.0008 (4)	−0.0021 (4)	−0.0059 (4)
C1	0.0191 (6)	0.0163 (6)	0.0209 (6)	0.0011 (5)	−0.0032 (5)	−0.0048 (5)
C2	0.0219 (6)	0.0229 (6)	0.0194 (6)	0.0010 (5)	−0.0007 (5)	−0.0058 (5)
C3	0.0204 (6)	0.0189 (6)	0.0191 (6)	0.0046 (5)	−0.0051 (5)	−0.0054 (5)
C4	0.0275 (7)	0.0217 (7)	0.0246 (7)	0.0050 (5)	−0.0084 (5)	−0.0114 (5)
C5	0.0258 (7)	0.0187 (6)	0.0311 (7)	−0.0003 (5)	−0.0093 (5)	−0.0075 (5)
C6	0.0219 (6)	0.0200 (6)	0.0270 (7)	−0.0009 (5)	−0.0021 (5)	−0.0030 (5)
C7	0.0227 (6)	0.0202 (6)	0.0221 (6)	0.0022 (5)	−0.0016 (5)	−0.0064 (5)
C8	0.0202 (6)	0.0155 (6)	0.0193 (6)	0.0033 (5)	−0.0053 (5)	−0.0046 (5)
C9	0.0211 (6)	0.0180 (6)	0.0202 (6)	−0.0061 (5)	0.0031 (5)	−0.0073 (5)
C10	0.0219 (6)	0.0259 (7)	0.0234 (7)	0.0000 (5)	−0.0051 (5)	−0.0081 (5)
C11	0.0194 (6)	0.0233 (7)	0.0273 (7)	0.0020 (5)	−0.0015 (5)	−0.0099 (5)
C12	0.0266 (6)	0.0158 (6)	0.0197 (6)	−0.0059 (5)	−0.0002 (5)	−0.0037 (5)
C13	0.0314 (7)	0.0206 (7)	0.0278 (7)	0.0010 (5)	−0.0120 (6)	−0.0062 (5)
C14	0.0228 (6)	0.0174 (6)	0.0336 (7)	0.0013 (5)	−0.0060 (5)	−0.0088 (5)
C15	0.0395 (8)	0.0260 (7)	0.0221 (7)	−0.0036 (6)	−0.0014 (6)	−0.0083 (6)

### Geometric parameters (Å, °)

**Table:**

O1—C1	1.3606 (14)	C7—C8	1.4072 (17)
O1—C9	1.4112 (14)	C7—H7	0.9500
N1—C2	1.3010 (16)	C9—C10	1.3765 (18)
N1—C3	1.3776 (16)	C9—C14	1.3774 (18)
N2—C1	1.2954 (15)	C10—C11	1.3894 (17)
N2—C8	1.3777 (15)	C10—H10	0.9500
C1—C2	1.4285 (17)	C11—C12	1.3877 (18)
C2—H2	0.9500	C11—H11	0.9500
C3—C4	1.4080 (17)	C12—C13	1.3898 (18)
C3—C8	1.4159 (17)	C12—C15	1.5057 (17)
C4—C5	1.3701 (18)	C13—C14	1.3860 (18)
C4—H4	0.9500	C13—H13	0.9500
C5—C6	1.4046 (18)	C14—H14	0.9500
C5—H5	0.9500	C15—H15A	0.9800
C6—C7	1.3717 (17)	C15—H15B	0.9800
C6—H6	0.9500	C15—H15C	0.9800
			
C1—O1—C9	117.73 (9)	C7—C8—C3	119.03 (11)
C2—N1—C3	116.77 (10)	C10—C9—C14	121.60 (12)
C1—N2—C8	115.51 (10)	C10—C9—O1	120.33 (11)
N2—C1—O1	121.46 (10)	C14—C9—O1	117.94 (11)
N2—C1—C2	123.85 (11)	C9—C10—C11	118.64 (11)
O1—C1—C2	114.69 (10)	C9—C10—H10	120.7
N1—C2—C1	121.82 (11)	C11—C10—H10	120.7
N1—C2—H2	119.1	C12—C11—C10	121.49 (11)
C1—C2—H2	119.1	C12—C11—H11	119.3
N1—C3—C4	119.73 (11)	C10—C11—H11	119.3
N1—C3—C8	120.60 (11)	C11—C12—C13	117.97 (11)
C4—C3—C8	119.67 (11)	C11—C12—C15	121.39 (11)
C5—C4—C3	120.09 (12)	C13—C12—C15	120.63 (11)
C5—C4—H4	120.0	C14—C13—C12	121.48 (12)
C3—C4—H4	120.0	C14—C13—H13	119.3
C4—C5—C6	120.34 (11)	C12—C13—H13	119.3
C4—C5—H5	119.8	C9—C14—C13	118.74 (11)
C6—C5—H5	119.8	C9—C14—H14	120.6
C7—C6—C5	120.65 (11)	C13—C14—H14	120.6
C7—C6—H6	119.7	C12—C15—H15A	109.5
C5—C6—H6	119.7	C12—C15—H15B	109.5
C6—C7—C8	120.23 (11)	H15A—C15—H15B	109.5
C6—C7—H7	119.9	C12—C15—H15C	109.5
C8—C7—H7	119.9	H15A—C15—H15C	109.5
N2—C8—C7	119.52 (11)	H15B—C15—H15C	109.5
N2—C8—C3	121.45 (11)		
			
C8—N2—C1—O1	−179.56 (9)	C6—C7—C8—C3	−0.28 (17)
C8—N2—C1—C2	−0.09 (17)	N1—C3—C8—N2	0.09 (17)
C9—O1—C1—N2	0.45 (16)	C4—C3—C8—N2	−179.94 (10)
C9—O1—C1—C2	−179.07 (10)	N1—C3—C8—C7	179.79 (10)
C3—N1—C2—C1	0.04 (17)	C4—C3—C8—C7	−0.23 (17)
N2—C1—C2—N1	0.07 (19)	C1—O1—C9—C10	−75.16 (14)
O1—C1—C2—N1	179.57 (10)	C1—O1—C9—C14	108.80 (12)
C2—N1—C3—C4	179.91 (10)	C14—C9—C10—C11	−2.43 (18)
C2—N1—C3—C8	−0.11 (16)	O1—C9—C10—C11	−178.32 (10)
N1—C3—C4—C5	−179.65 (10)	C9—C10—C11—C12	0.18 (18)
C8—C3—C4—C5	0.37 (18)	C10—C11—C12—C13	1.90 (18)
C3—C4—C5—C6	0.00 (18)	C10—C11—C12—C15	−178.87 (11)
C4—C5—C6—C7	−0.53 (19)	C11—C12—C13—C14	−1.86 (18)
C5—C6—C7—C8	0.67 (18)	C15—C12—C13—C14	178.91 (11)
C1—N2—C8—C7	−179.68 (10)	C10—C9—C14—C13	2.47 (18)
C1—N2—C8—C3	0.02 (16)	O1—C9—C14—C13	178.45 (10)
C6—C7—C8—N2	179.42 (10)	C12—C13—C14—C9	−0.27 (18)
